# What do patients with a rare cancer living in rural, regional or remote areas and stakeholders want from a peer support program? A qualitative study

**DOI:** 10.1186/s12885-025-13782-0

**Published:** 2025-02-25

**Authors:** L. Hemming, S. F. A. Duijts, C. Cockburn, C. Wilson, E. Y. N. Yuen, E. Spelten

**Affiliations:** 1 Violet Vines Marshman Centre for Rural Health Research, Rural Health School, La Trobe University, Bendigo, VIC 3552 Australia; 2 Department of Research and Development, Netherlands Comprehensive Cancer Organisation (Integraal Kankercentrum Nederland, IKNL), Utrecht, The Netherlands; 3 Department of Medical Psychology, Amsterdam University Medical Centres, Location Vrije Universiteit, Amsterdam, The Netherlands; 4 Rare Cancers Australia, Bowral, New South Wales Australia; 5 Melbourne School of Population and Global Health, Melbourne University, Melbourne, Victoria Australia; 6 School of Nursing and Midwifery, Deakin University, Geelong, Victoria Australia; 7 Monash Health, Clayton, Victoria Australia

**Keywords:** Cancer, Rare cancer, Peer support, Rural, Regional and remote health

## Abstract

**Background:**

Patients with a rare cancer in rural, regional, and remote Australia experience heightened challenges in their illness journey, including significant psychosocial impacts. Although peer support has shown benefits for common cancer patients living in urban areas, these programs often do not reach underserved groups for instance those with a rare cancer, or those living in rural, regional or remote areas. This study aimed to explore the characteristics of peer support programs for patients with a rare cancer living in rural, regional or remote areas.

**Methods:**

Focus groups and interviews were conducted with 39 people with a rare cancer and 10 healthcare providers to explore key points for inclusion in a peer support service for people diagnosed with a rare cancer living in rural, regional or remote areas. Data were transcribed verbatim and analysed thematically, using Nvivo.

**Results:**

Participants described their peer support needs using the key terms who, what, how, where, and when. Participants advocated for a flexible, multicomponent intervention that could meet the varied and fluctuating needs of this group. Participants also noted challenges with the practical delivery of such a service, specifically, the risk of receiving misinformation, adverse emotional reactions, interpersonal challenges and implementation issues.

**Conclusions:**

This study highlights the role of peer support in addressing unmet needs of patients with a rare cancer, particularly in rural areas, emphasising the importance of tailored, flexible, and multimodal interventions for the delivery of peer support that addresses diverse needs.

**Supplementary Information:**

The online version contains supplementary material available at 10.1186/s12885-025-13782-0.

## Introduction

Rare cancers are defined as those with an incidence rate of fewer than 6 cases per 100,000 people annually [1]. Despite being labelled ‘rare’, they encompass over 220 tumour types and collectively account for one in five cancer diagnoses [2]. Some examples of tumour types considered rare are head and neck cancers, sarcomas and neuroendocrine cancers. Patients with rare cancers often face a more challenging illness trajectory than those with common cancers, including delays in diagnosis, incorrect treatments, and limited access to clinical trials [3, 4]. Furthermore, the 5-year survival rate for individuals with rare cancers is significantly lower at 52%, compared to 69% for those with common cancers [5]. In a recent systematic review, it was reported that patients with a rare cancer experience various adverse psychosocial outcomes following diagnosis, including depression, anxiety, suicidality, and post-traumatic stress disorder (PTSD) [6]. Also, patients with a rare cancer were reported to have a higher risk of PTSD and suicidality compared to those with common cancers [6].

In addition to the significant challenges patients with a rare cancer face following their diagnosis, patients living in rural, regional or remote areas also struggle to access clinical, psychological, and informational support [7]. Recent data suggest that cancer patients residing far from metropolitan centres are at a higher risk of dying within five years of diagnosis [8]. Similar to patients with a rare cancer, rural cancer patients encounter numerous obstacles when accessing care, including limited availability of treatments and support services, transportation barriers, financial difficulties, and restricted access to clinical trials [9]. Qualitative research suggests that many unique psychosocial needs of rural cancer patients remain unmet, particularly in the domains of financial help, travel support, information needs, accessing care and connection with others [7, 10, 11].

One approach frequently used to address psychosocial needs of cancer patients is provision of peer support [12]. Peer support is defined as ‘a system of giving and receiving help founded on key principles of respect, shared responsibility, and an agreement of what is helpful’ [13] (p. 6). A peer, in this context, is defined as ‘an equal, someone with whom one shares demographic or social similarities’, and support refers to ‘the kind of deeply felt empathy, encouragement and assistance that people with shared experiences can offer one another within a reciprocal relationship’ [14] (p. 1). Peer support can take many forms including structured or unstructured approaches; group or individual services; in-person or telehealth meetings; and can occur with a range of frequencies, with individual sessions varying in duration [15–17]. Peer support may be led by either trained professionals or peers themselves [12, 18], often those who share a diagnosis with the person being supported [33].

Peer support for cancer patients has many benefits. Patients often seek emotional insights and advice on coping from those who have had similar experiences, thereby enhancing their preparedness and emotional resilience [19, 20]. Peer support groups provide structured platforms for individuals to share experiences and offer mutual emotional support [12, 21]. This approach is rooted in patient and community empowerment and complements traditional psychosocial care [18, 22], which is paramount in the context of rural residents who may struggle to access healthcare required. Participation in these groups can combat isolation, provide access to diverse coping strategies, and empower individuals through shared experiences [18, 23, 24]. Although the benefits of peer support for cancer patients are clear, challenges, such as navigating emotions and discerning the reliability of information, underscore the need for structured, quality peer support integrated into broader cancer care initiatives [24].

Despite the unique psychosocial needs of patients with a rare cancer living in rural, regional and remote areas, and despite the proven benefits of peer support for cancer patients, existing peer support services tend not to reach underserved communities including those diagnosed with a rare cancer living in rural regional or remote areas [25]. The present study therefore aimed to explore the characteristics of peer support programs for patients with a rare cancer living in rural, regional or remote areas. Specifically, the research questions addressed were: (1) ‘what do patients with a rare cancer living in rural, regional or remote areas and stakeholders want from a peer support program?’ and (2) ‘what are the perceptions of healthcare providers about the feasibility of achieving such a peer support program?’

## Methods

### Participants and recruitment

Two groups of participants were recruited: people with rare cancer living in rural, regional or remote areas and those providing services to this group (healthcare providers). Although the primary focus was on gathering insights from those who would ultimately benefit from a peer support service, the perspectives of relevant healthcare providers (e.g., medical specialists and supportive care workers working with this patient group) were also considered valuable to identify potential feasibility and implementation challenges of any peer support service proposed for operation within the existing health system. Sample size was determined using information power, a concept based not on numerical size alone, but on the richness and relevance of the data in addressing the research question. Information power is influenced by several factors including the specificity of the sample, the study aims, the strength of the theoretical framework, the quality of dialogue and the chosen analytical strategy [26]. Given the study’s focused objectives and targeted participant group, the sample size was deemed sufficient to generate meaningful insights.

#### People with rare cancer

People with rare cancer were eligible for inclusion if they: (i) had been diagnosed with a rare tumour type in the past 5 years, (ii) lived in a rural, regional or remote location (areas defined using the Modified Monash Model as regional centres, small, medium & large rural towns, remote communities and very remote communities), (iii) were aged 18 years or over, (iv) were comfortable with the English language, and (v) did not self-identify as having a serious psychiatric illness. People with rare cancer were recruited with the help of partner organisation Rare Cancers Australia (RCA). RCA is the largest patient platform in Australia, connecting with roughly 85 patients with a rare cancer a month. RCA assisted with recruitment by contacting potentially eligible participants through: (i) a database of registered users, and (ii) official RCA social media channels. Interested participants were asked to contact the research team, from whom they were directed to an online questionnaire which included a participant information sheet, eligibility questions, a consent form and demographic questionnaire. Purposive maximum variation sampling was used to recruit participants that varied on the key dimensions of age and gender.

#### Healthcare providers

Participants were eligible for inclusion if they: (i) had worked with at least one rare cancer patient, in a rural regional or remote setting, in the past 5 years, and (ii) were comfortable communicating in the English language. These participants were recruited via existing networks of both the research team, and the advisory group for the project. Potential participants were invited to complete an online questionnaire which included a participant information sheet, eligibility questions, consent form and demographic questionnaire. Purposive maximum variation sampling was used to recruit participants that varied by age, gender and job title. We sought an even split across a diverse range of job titles in order to capture the views of healthcare professionals working across the spectrum of the entire healthcare ecosystem.

### Data collection and procedure

#### People with rare cancer

Once eligible participants had completed the online questionnaire, which included a consent form, they were invited to participate in one of several pre-scheduled focus groups, or alternatively were asked to contact the research team with availability for a one-to-one interview. Both focus groups and interviews were deemed appropriate due to the ability to enhance data richness, offer iterative and contextual insights and increase trustworthiness [27, 28].

Six focus groups were conducted, with each group comprising between three and ten participants. Additionally, four individual interviews were conducted. Focus groups lasted on average 1 h and 10 min whilst interviews lasted on average 35 min. Both focus groups and interviews were conducted online using teleconferencing software Zoom and were led by LH. Focus groups and interviews were audio recorded using Zoom functions and were transcribed verbatim. The transcription was de-identified, and each statement was assigned a unique number due to the large number of participants and the inability to determine individual speakers from the audio recording. Focus groups and interviews followed a semi-structured format with questions focussing on participants’ prior experience with peer support, as well as key points for inclusion in a future peer support service (see Appendix A for the topic guide). Following participation, participants were compensated for their time with a $40 voucher, a rate in line with VCCC Alliance guidelines [29].

#### Healthcare providers

After completing the online consent form, participants were contacted to arrange a one-to-one interview with LH. Interviews were conducted online via teleconferencing software Zoom, and were audio recorded then transcribed verbatim. Interviews lasted on average 36 min. Semi-structured interviews were conducted with a topic guide that focussed on participants knowledge of, experiences with, and opinions about peer support for patients with a rare cancer living rurally and remotely as well as key points for inclusion in any future service (see Appendix B for the topic guide).

### Data analysis

An inductive thematic analysis was conducted on the data using Nvivo [30]. All interview data were analysed together to identify areas of convergence and divergence. To conduct thematic analysis following Braun and Clarke’s 6-step process [31, 32], the researcher first familiarised herself with the transcripts by reading and re-reading them, noting initial ideas. Next, initial codes were generated systematically across the entire dataset, organising data into meaningful groups. In the third step, themes were searched for by collating codes into potential themes and gathering all relevant data for each potential theme. In the fourth step, these themes were reviewed as a team, checking if they worked in relation to the coded extracts and the entire dataset, generating a thematic map of the analysis. The themes were then defined and named, refining the specifics of each theme and the overall story the analysis tells, ensuring clear definitions and names for each theme.

## Results

### Sample characteristics

#### People with rare cancer

A total of 39 people with rare cancer were recruited (see Table 1 for participant characteristics). The majority of participants were male (67%), and the average age was 35.3 (SD = 14.5). 28% of participants identified as Aboriginal, 5% as Torres Strait Islander, 18% as Aboriginal and Torres Strait Islander and 49% as neither. Most of the sample were working either part time or full time, and over half were married or partnered. A range of rare cancer diagnoses were represented with nearly a third of participants in the post-treatment phase of their cancer journey.

**Table 1:** Patient participant demographic information

	*N*	%
Gender		
- Male	26	67%
- Female	13	33%
- Non-binary	-	-
Age (Mean, SD)	35.3	14.5
Aboriginal and Torres Strait Islander		
- Aboriginal	11	28%
- Torres Strait Islander	2	5%
- Aboriginal & Torres Strait Islander	7	18%
- Neither	19	49%
Employment status		
- Working full time	12	31%
- Working part time	23	59%
- Working casually	1	2%
- Not working	3	8%
Marital status		
- Single	14	36%
- Married / partnered	24	61%
- Separated / divorced	-	-
- Widowed	1	3%
Cancer diagnosis		
- Breast cancer	1	2.5%
- Cancers of the endocrine organs	1	2.5%
- Digestive cancers	7	18%
- Female genital cancers	1	2.5%
- Head and neck cancers	4	10%
- Haematological cancers	6	15%
- Male genital and urogenital cancers	1	2.5%
- Neuroendocrine tumours	1	2.5%
- Sarcomas	12	31%
- Skin cancers and non-cutaneous melanoma	3	8%
- Thoracic	1	2.5%
- Not reported	1	2.5%
Stage of cancer journey		
- Recently diagnosed	3	8%
- In active treatment	12	31%
- Post treatment	16	41%
- In remission / no evidence of disease	7	18%
- Experiencing a recurrence	4	10%
- Advanced stage	11	28%
- Stable	8	21%
- Other	-	-

#### Healthcare providers

Ten healthcare providers were recruited (see Table 2 for participant characteristics). There was an even split between males and females and participants were aged 40.6 years (SD = 8.6) on average. Participants included medical specialists, registered nurses, care co-ordinators and supportive care workers. On average, healthcare providers had been working with cancer patients for approximately 9.5 years (range 3 to 20 years).

**Table 2:** Healthcare provider demographic information

	*N*	%
Gender		
- Male	5	50%
- Female	5	50%
- Non-binary	-	-
Age (Mean, SD)	40.6	8.6
Aboriginal and Torres Strait Islander		
- Aboriginal	-	-
- Torres Strait Islander	-	-
- Aboriginal & Torres Strait Islander	-	-
- Neither	10	100%
Role		
- Medical specialist	3	30%
- Registered nurse	2	20%
- Care coordinator	3	30%
- Supportive care worker	2	20%
Use a language other than English at home?		
- Yes	1	10%
- No	4	40%
- Missing	5	50%
Born in Australia?		
- Yes	5	50%
- No	-	-
- Missing	5	50%
Experience working with people with cancer (Mean years, range)	9.5	3–20

### Key findings

Six themes were identified through thematic analysis. Five themes related to the **who**, **what**, **how**, **where** and **when** of peer support. Taken together, these themes described a hypothetical ‘ideal’ peer support service for patients with a rare cancer living in rural, regional and remote Australia as perceived by both people with a rare cancer and healthcare providers. In one additional theme, the practical **challenges** associated with delivering such a service were identified. Each theme is discussed in more detail below.

#### Who is a peer?

Participants engaged in rich debate about exactly who was considered a peer in the context of patients with a rare cancer living in rural, regional and remote areas. Some participants felt that having the same, or a similar diagnosis, was important because these individuals were likely to have had a similar experience and be relatable. Others felt that the specific diagnosis was not important, but that a peer should have a diagnosis that falls under the umbrella of a rare cancer diagnosis. This was because participants recognised the more difficult trajectory faced by a rare cancer patient and indicated that hearing from somebody with any rare cancer diagnosis would be useful. Experience with similar forms of treatment was also described as defining a peer. Healthcare providers added that grouping people with similar diagnoses or treatment pathways could help to prevent people experiencing unrealistic expectations due to differing illness trajectories and treatment options.

*“So if you have someone that goes to the same treatment*,* you tend [to] listen to whatever they’re gonna say to you*,* because you guys share the same diagnosis.” (Patient 23*,* Focus group 4)*.

*“I just think it would be beneficial*,* with peer support to have people of similar cancers or similar treatment pathways. I think that’s where some of the confusion lies is when they go*,* oh yes*,* but I spoke to somebody that had this certain type of cancer and they’re fine now and XYZ and then it looks different for them…” (Healthcare provider 9*,* Female*,* 30*,* Care coordinator)*.

Participants (both patients and healthcare providers) also described the phase of illness as relevant to finding the right cohort. Most participants wanted a person to support them who was further along in their cancer journey, so that they could advise them of what lies ahead, and even encourage a sense of hope and reassurance.

*“…Because I need the experience. I need their treatment advice. I would also need a survivorship (sic.) in the group. That will help me and give me hope. That ‘oh he was once scared but he’s now okay’ ‘oh he’s now getting rid of it’.” (Patient 5*,* Focus group 2)*.

Other participants (including both patients and healthcare providers) were less focussed on disease characteristics and instead felt it was important that peers were matched on demographic variables or life experiences. For instance, some participants felt that it was important that a peer was the same gender, age or culture. Others wanted to connect with peers who had children, or with peers that had similar interests to them.

*“… people generally wish to connect on one of two things*,* they want to speak with someone who’s had a…similar diagnosis and treatment. Or they might say I… just want to speak with someone who had kids in primary school. So*,* I might [ask]…whether they think it might be more helpful to speak with someone who’s been through something clinically quite similar or socially similar.” (Healthcare provider 8*,* Female*,* 32*,* Supportive care worker)*.

*“There is a group in my local town. But it is a lot of older people*,* so I don’t attend that usually. And*,* you know*,* not to sound horrible but they just don’t have the same sort of things and problems that I have*,* so I just don’t find it that helpful.” (Patient 29*,* Focus group 5)*.

All participants acknowledged that forming a meaningful connection with peers could be challenging and could not be predicted based on demographic factors or life experiences alone. Participants instead often spoke about the traits they perceived as being important in a supportive peer. These were characteristics that transcended all life experiences, whether clinical or social. Traits included empathy, sympathy, being understanding, being able to navigate support, cultural sensitivity and the ability to create a safe and comfortable environment.

*“The bigger problem is that*,* what you want is peers that form a meaningful connection… it is quite possible that the man with prostate cancer and the young woman with thyroid cancer could hit off completely*,* because I don’t know*,* they’ve got a love of the Matilda’s*,* you know… The problem is that it’s very difficult to predict who’s going to click.” (Healthcare provider 2*,* Male*,* Age not reported*,* Medical specialist)*.

*“…need to have some…empathy and sympathy and…compassion and be a good ear…for listening…we all like to offload all our feelings… I guess for me*,* it would…be just being able to offload and have that understanding*,* I suppose of where I’m coming from.” (Patient 4*,* Focus group 2)*.

As well as discussing **who** they would consider to be a suitable peer, patients spoke about the reciprocal nature of peer support, and spoke about the experience of, or desire to, *provide* peer support to others. Patient participants spoke about wanting to provide peer support for altruistic reasons, highlighting that they wanted to give the support to others that they had needed and received themselves throughout their journey.

*“So*,* I think*,* sometimes it can be very*,* very good for people and I think I was giving a lot back because I’ve been through a lot more treatment probably than a lot of my current peer groups that I know about in the Australian groups at least. And*,* I can give that information about my treatment and all that that sort of thing*,* which can be good.” (Patient 29*,* Focus group 5)*.

Participants, both patients and healthcare providers, were cognisant, however, of the toll that providing peer support could have on them. They frequently spoke about ensuring boundaries were put in place, acknowledging that it can take time for an individual to be in a place where they are ready to support another, and that this was a fluctuating headspace.

*“You have to find a way to match people with where they’re at in their journey. I’m more able to talk about it now. I couldn’t say the C word for about a year. And maybe sort of at the 6-month to 18-month mark I wouldn’t have been able to talk to other people about it. I’m in a different place now but I know that that’s not static either.” (Patient 31*,* Focus group 5)*.

#### What is the preferred content of a peer support program?

Participants had many ideas about the possible focus and content of a peer support program. Participants suggested that the most important aspect of a peer support program was providing an opportunity to learn through experiential knowledge obtained from others. People with a rare cancer and healthcare providers alike acknowledged that peer support allows patients to learn ‘tips and tricks’ from those who have been through this journey, with these tips generally not available to them from clinicians. Specifically, participants wanted to learn strategies for dealing with medication or treatment side effects, practical solutions to problems such as debt and travelling to appointments and how to deal with family and friends.

*“They also offer practical advice like dealing with challenges of cancer such as finding financial assistance.” (Patient 36*,* Interview)*.

*“I think you find also that peer support is where you get some very practical advice that health professionals don’t necessarily always have as easily. It’s along the lines of I am completely making this up*,* but*,* if you mix your resource with apple juice rather than water*,* that’s really helping. Do you know what I mean? We health professionals never drink the resource. We probably should*,* but we don’t.” (Healthcare provider 2*,* Male*,* Age not reported*,* Medical specialist)*.

Partnered with this experiential knowledge, patient participants also wanted to receive more information about their diagnosis, treatment options and their cancer journey. Though participants favoured the delivery of this information in a peer support setting, they wanted this information to come from a trained healthcare professional as opposed to the experiential knowledge imparted by peers.

*“And then*,* once a week*,* sometimes once in 2 weeks*,* the group is led by a medical doctor and they always give advice on how to manage our cancer… Mostly it’s about the emotion*,* how to open your emotions*,* your feelings. And secondly is like a reminder how to take your medications and what you need to do medically to keep fit.” (Patient 6*,* Focus group 2)*.

Alongside seeking information and tips, patient participants expressed a strong need for emotional support from a peer support program. One of the main functions of a peer support program was described as facilitating relationships with others. This need was described as particularly important for people with a rare cancer, who often lacked opportunities for unplanned interactions due to their unique treatment regimens. Participants wanted a space where they could share their troubles and receive emotional support from others who had undergone similar experiences. Knowing that others had faced similar challenges was described as comforting.

*“It’s like an observational thing more than a like ‘I’m gonna get involved with your life and guide you’*,* kind of thing. So it’s more around*,* ‘I’m feeling like this’ and you kinda going*,* ‘yeah*,* that’s normal’. You know or going*,* ‘that sucks’*,* you know*,* ‘that’s terrible’” (Healthcare provider 6*,* Female*,* 48*,* Supportive care worker)*.

*“I’m doing this Cancer Council meditation class and the class was nice*,* but then they would put us into these breakout rooms where we would communicate with each other. And we’re supposed to talk about how the meditation bloody went. But I think everybody in there was just like*,* ‘hello other people with cancer!! What’s yours??” And that was the best bit. It was feeling that we weren’t alone in it.” (Patient 3*,* Focus group 1)*.

*“It’s just about having a platform where [patients with a rare cancer] can feel connected to other people*,* talk to other people about what they’re going through. You know*,* get that off their chest.” (Healthcare provider 10*,* Male*,* 34*,* Supportive care worker)*.

#### How should peer support be delivered?

There was little consensus on the delivery format for peer support, both within participant groups (patients and healthcare providers) and across participant groups. Participants had mixed views on whether peer support should be delivered in a group setting or on an individual basis. Group settings were noted to open patients up to a diverse array of experiences, which was described as beneficial. Group settings also meant that the emphasis went beyond talking to listening, and this appealed to many. Conversely, participants reported that individual settings might encourage more open communication because of less concern about anonymity and privacy and this, in turn, could lead to greater rapport than in a group setting.

*“…I prefer the group. Because [in] the group we have people with diverse information*,* diverse history*,* diverse professions when it comes to cancer management and treatment… if you have [an] individual*,* it’s not going to give you a wider knowledge… There must be variation*,* so I prefer groups.” (Patient 8*,* Focus group 2)*.

*“I think with rare cancers*,* I think one-to-one is probably preferable as it is*,* perhaps*,* just that ability to really ask more in-depth questions of someone. And to establish rapport over time*,* people might find that helpful.” (Healthcare provider 8*,* Female*,* 32*,* Supportive care worker)*.

There were mixed views on whether peer support groups should be structured or unstructured. The main benefit of structured sessions was that they encouraged participation from everyone. However, participants noted that unstructured sessions gave patients a greater sense of power and control, allowing for more free-flowing and natural conversations.

*“I think you need a little bit of structure*,* otherwise people won’t go. They’ll sort of get a bit intimidated and feel like they have to speak.” (Healthcare provider 5*,* Male*,* 52*,* Care coordinator)*.

*“I think I would like it to be unstructured so that members of the group will be able to ask questions and based on their questions*,* you know*,* topics can be raised.” (Patient 6*,* Focus group 2)*.

*“Yeah kind of a mix*,* like… forecast in advance…in 2 weeks we’re going to be talking about…how to deal with family. So then you could make the effort to call in…if that’s a particular thing that resonates with you then that’s the that’s the group you’re not going to miss or you’ll make sure you can call in from work…” (Patient 31*,* Focus group 5)*.

Participants had differing opinions on the optimal frequency of meeting and number of peer support sessions. Preferences for frequency of peer support ranged from weekly to quarterly, and preferences for length of the intervention ranged from three sessions to ongoing.

*“It really depends on the day and week or your mood*,* or just where you’re at basically. For me*,* when I was first diagnosed like I didn’t have time between appointments and trying to balance my working life and my normal life*,* like I didn’t have time*,* so I didn’t connect with anyone… But other people might want it every week*,* every day…” (Patient 29*,* Focus group 5)*.

Most participants agreed that peer support that took place in a group setting should be moderated or facilitated. However, there was disagreement about who was best placed to moderate these sessions. Although some suggested that it was important that healthcare professionals lead groups, believing their clinical expertise could prevent the spread of misinformation, others contended it would be more appropriate for a patient to lead the group, notwithstanding the sustainability challenge of this approach. A third suggestion was that carers might be well placed to lead peer support groups. Regardless of the facilitator’s identity, participants strongly advocated facilitator training, particularly because of the need for sensitivity in many of the topics covered.

*“I think that moderation is so important and particularly when people are early diagnosed… to have a bit of a sanity checking aspect… guardrails…when people are very vulnerable… you do need someone to stop people [talking]… about the final stages of your relatives’ death…. [I need] for someone to go ‘that’s great*,* no we’re not doing that’*,* you know…” (Patient 31*,* Focus group 5)*.

Ultimately, participants wanted a peer support service that was flexible to meet varied and fluctuating needs. Participants acknowledged that peer support could offer them different things at different stages, and that needs and wants were not static. Therefore, a flexible model of delivery that suited different styles of communication was viewed as desirable.

*“Mixed kind of modes of [delivery]…depending how you feel*,* but also mix[ed]…conversations…sometimes you don’t even want to talk about cancer you just wanna be in a safe space with people who are having a crappy day just like you. But just talk about a book or have a coffee or whatever. Definitely the option to have all different platforms and different modes would be really helpful*,* but I know that’s also challenging for distance and you know people’s locations and work commitments*,* etc.” (Patient 29*,* Focus group 5)*.

Aside from the specific details of how peer support should be delivered, participants generally agreed that peer support should include the provision of a safe space to share experiences with others. In fact, participants spoke of peer support as being like a ‘family’ or a ‘home away from home’.

#### Where should peer support take place?

There were mixed opinions on whether peer support should take place online, over the telephone, in person or a combination of all three. Many participants (both patients and healthcare providers) acknowledged that although their preference would be for an in-person peer support service, for pragmatic reasons, for instance the broad geographic diversity of rural patients, and the infrequent prevalence of rare cancers, online or telephone delivery was probably more appropriate.

*“Preferably*,* in person face to face*,* but I think for a rare cancer in rural setting*,* you’re not gonna do that. It won’t be a rare cancer. It will be prostate or breast or bowel.” (Healthcare provider 5*,* Male*,* 52*,* Care coordinator)*.

In addition to pragmatism, participants spoke about online and telephone formats bringing other benefits for instance allowing distance and anonymity as well as removing practical limitations such as the number of people able to be in one ‘room’ at any one time. These benefits were felt keenly during the pandemic, when patients with a rare cancer were extremely isolated due to social distancing requirements. Participants also spoke about generally preferred methods of communication.

*“So there needs to be some sort of virtual and online element to that. I think depending on your day you like to communicate differently but also different generations communicate differently.” (Patient 31*,* Focus group 5)*.

Despite this, participants, particularly healthcare providers, raised concerns about the online format, including internet accessibility and computer literacy issues. Patients wanted a combination of in-person and online formats, to allow greater accessibility and flexibility for everyone.

*“…Online*,* that’s one of the challenges if you ask me. It’s not going to be easy. I see a lot of that here. People don’t have a good internet literacy*,* let’s say*,* or online literacy. So that’s something that people will struggle with here” (Healthcare provider 7*,* Male*,* 42*,* Medical specialist)*.

#### When do people want to receive peer support?

Both patient and healthcare provider participants believed that peer support would be beneficial at all stages, including pre-diagnosis, diagnosis, treatment, post-treatment, and advanced palliative care. They particularly recognised the emotional toll of receiving a cancer diagnosis, especially a rare one, highlighting the need for peer support during this time. Nonetheless, they emphasised that peer support would be valuable throughout all stages of the cancer experience.

*“So I feel that peer group is something that [is] important to everybody at any time or at any stage.” (Patient 28*,* Focus group 4)*.

Conversely, some patient participants felt that receiving peer support at the time of diagnosis was *un*helpful, noting that at this stage you could be in ‘panic’ or ‘crisis’ mode. At this time, it was not necessarily deemed beneficial to hear from others what lies ahead, as this was described as too overwhelming.

*“Some of them are at the start of the process and I think that some of them don’t want to face what’s coming*,* like in detail.” (Healthcare provider 6*,* Female*,* 48*,* Supportive care worker)*.

Participants also spoke about the timing of peer support interactions, emphasising that these should be accessible to as many people as possible.

*“I don’t find a lot of commonality I guess for what I’m going through. But it’s Wednesday lunchtime. So*,* when I first got diagnosed*,* I had a child who wasn’t even in preschool then*,* but then I had a preschooler. And so I’m not calling into a support group during the day at lunch time. And also difficult at work*,* but then I know that probably works for a lot of other people.” (Patient 31*,* Focus group 5)*.

#### What are the challenges of a peer support program for patients with a rare cancer living in rural, regional and remote Australia?

The previous five themes describe participants perceptions of the ideal, gold-standard peer support service as envisaged by both rural patients with a rare cancer and healthcare providers. However, participants also identified several challenges to delivering such a service. One of the most common issues raised by both patients and healthcare providers was the possibility of misinformation being shared in a peer support group. Participants were particularly concerned about being told about treatments that were outdated or not based on scientific evidence. These participants strongly advocated for peer support sessions to be moderated by a trained medical professional to minimise the amount of misinformation shared.

*“…a lot of the information that is peddled on internet forums or peer groups should be taken with a grain of salt because it often lacks scientific rigour. And scientific rigour doesn’t come from people like you and me exchanging information about a particular drug or not*,* but can only really be obtained from a trained doctor or cancer specialist or pathologists or geneticists*,* or some of those people. Everything else is just hearsay.” (Patient 20*,* Focus group 3)*.

Participants also expressed concerns about being exposed to others’ ‘horror stories’. More broadly, participants were worried about the emotional impact of hearing others’ cancer journeys, and whether this would help or hinder their own emotional state. Healthcare providers emphasised the importance of follow-up care with patients who become distressed as a result of peer interactions.

*“I don’t bring up the horror stories. Because it’s terrifying. You know when you’re staring at this long treatment plan and based on TV and whatever else…you kind of go like I don’t wanna know about it. Like*,* until it happens to me*,* I don’t wanna know about it because… it doesn’t happen to everyone*,* the horror story stuff.” (Healthcare provider 6*,* Female*,* 48*,* Supportive care worker)*.

*“Some topic points can be triggering for some patients and they might get a bit overwhelmed and upset… Our best practice is to ensure we check in with the patient after the support group*,* give them a phone call and just support them… You know*,* just wanted to check in and talk this out*,* see how you feel and if you have any concerns.” (Healthcare provider 10*,* Male*,* 34*,* Supportive care worker)*.

On the other end of the spectrum, participants (both patient and healthcare provider) spoke about the challenges of hearing others’ success stories, which could raise unrealistic hopes. For example, many online peer support groups are worldwide, and so patients might get their hopes up about a treatment that is not available in their place of residence.

*“A lot of people in this group have done treatment of other sorts overseas and when I’ve looked at what Australia’s got in comparison to the same treatment*,* there’s only just like one centre*,* for instance*,* that’s just being built in South Australia but they won’t be taking any patients in for 18 months.” (Patient 30*,* Focus group 5)*.

Patient and healthcare provider participants also described a range of interpersonal problems that could arise, particularly in group settings. For instance, some raised the possibility that social dynamics within the group might make some individuals feel uncomfortable, and others might lack confidence when speaking in a group setting. Even one-on-one support could lead to problems, specifically an over-reliance on the peer support worker. Participants also described concern about losing group members to cancer, which could ultimately make them feel worse.

*“Sometimes I feel like there is this feeling of exclusion [when]… other people are actually talking about a certain situation whereby you don’t have any experience about or maybe this discussion has been going on for a very long time…you start feeling like you don’t really have any better business in the space.” (Patient 19*,* Focus group 3)*.

*“The other risk is loss…we’re gonna lose people because that’s the community that we’re in. So I think…a buddy system probably is very risky for people emotionally*,* especially given we’re not necessarily [professionals] or anything like that. So I think*,* again*,* moderators*,* guardrails*,* there’s supporting people but you know*,* you’ve got to be very realistic…” (Patient 31*,* Focus group 5)*.

Participants, particularly healthcare providers, had concerns about the acceptability of, and need for, a peer support intervention for patients with a rare cancer living in rural, regional and remote Australia. Specifically, participants showed some scepticism about the level of interest in participating in an intervention like this, noting that due to the rarity of the disease, it could be difficult to engage people with a rare cancer in such an intervention. Moreover, it was noted that those living in rural, regional and remote locations are less likely to seek support, and this again may inhibit uptake to such an intervention. More broadly than this, participants acknowledged that peer support simply ‘isn’t for everyone’, and that cancer patients are often very preoccupied with other medical appointments and this may mean they have little interest in a peer support program.

*“So*,* for some they really like [peer support] and others they don’t have the time or the energy…” (Healthcare provider 1*,* Female*,* Age not reported*,* Care coordinator)*.

Both people with a rare cancer and healthcare providers had concerns around the feasibility of delivering peer support. Participants noted that, currently, the peer support groups that *do* exist tend to be poorly advertised and promoted and are not visible to most patients. They advocated for more widespread promotion of future programs to help overcome challenges of attendance. Participants also expressed concern about the difficulties of recruiting peer support workers, and the welfare of these workers, both of which combined led to issues with the longevity of a peer support program. Participants encouraged top quality training and support for peer support workers to protect against burnout.

*“Best [facilitator] would be a consumer so a patient who’s been through it. But I think it’s unlikely*,* you know*,* they’re really rare. And if you had to rely on that for every support group*,* you probably wouldn’t have any… Because it’s a big job…Big enough job having cancer*,* let alone a rare one*,* or let alone running a support group.” (Healthcare provider 5*,* Male*,* 52*,* Care coordinator)*.

## Discussion

### Main findings

This study aimed to explore the characteristics of peer support programs for patients with a rare cancer living in rural, regional and remote areas. Both healthcare providers and people with rare cancer spoke about the who, what, how, where and when of peer support, as well as the challenges associated with service provision. A summary of key learnings and implications of these is provided in Table 3.

**Table 3:** Key learning points and implications for peer support for patients with a rare Cancer living rurally

Theme	Key Learning Points	Implications for Practice
Who is a Peer?	Patients define peers based on diagnosis, treatment experience, demographics, and shared life experiences.	Peer support programs should allow for flexible matching based on patient preferences, not just diagnosis.
What Support is Needed?	Patients value both emotional support and practical advice on treatment, side effects, and daily challenges.	Peer support should combine experiential knowledge sharing with access to verified medical information.
How Should Support be Delivered?	Preferences vary between structured vs. unstructured, individual vs. group formats.	Programs should offer a mix of formats, including structured groups, informal discussions, and one-on-one connections.
Where Should Support Take Place?	Online and telephone support improve accessibility, but in-person support is still valued.	Hybrid delivery models should be explored to ensure accessibility for rural patients while maintaining personal connections.
When is Support Most Needed?	Needs fluctuate across the cancer journey, particularly at diagnosis and major treatment decision points.	Support services should be flexible and available throughout different stages of the cancer journey.
Challenges of Peer Support	Risks include misinformation, emotional distress, and difficulties in maintaining engagement.	Programs should include training for peer facilitators, moderation, and structured support to ensure sustainability and safety.

Participants highlighted various factors important in defining a peer, including diagnosis, treatment, demographic similarity, and shared life experience. This aligns with existing literature, in which peers are defined as ‘an equal, someone with whom one shares demographic or social similarities’ and peer support is described as ‘understanding another’s situation empathically through the shared experience of emotional and psychological pain.’ [14] (p. 1). Previous research has also indicated that cancer patients prioritise a shared cancer diagnosis when identifying peers [33]. However, in the present study a number of participants indicated that simply sharing a diagnosis did not automatically make someone a peer. This suggests that identifying peers who might support a rare cancer survivor requires a more tailored, patient-centred approach and that further research could explore the variables that define peer status and how these vary between people and across time. Consistent with previous definitions of peers in the literature, participants in our study emphasised the importance of reciprocity in peer support, valuing both the opportunity to give and receive support [13, 14, 21].

Participants discussed peer support as a valuable source of education and learning, highlighting the unique ability of peers to foster experiential learning. This aligns with previous studies conducted with patients [12, 34], and is recognised as a key component of peer support more broadly [21, 35]. Participants suggested that the need for information was likely to be more acute for patients with a rare cancer than for more common cancer patients because of the lack of information and resources available for most rare cancer survivors. Indeed, in previous research, it has been confirmed that the most frequently reported unmet needs for patients with a rare cancer fall within the information domain [3]. This suggests that peer support programs should consider including the provision of information, beyond that provided through peers sharing their experiences. Beyond seeking information, both patients and healthcare providers acknowledged that peer support can provide emotional support and connection with others. This finding is consistent with previous research on patients in the cancer field [12, 22] and aligns with broader models of peer support [18, 36], highlighting the need for peer support models to emphasise the importance of social connection and emotional support.

Participants expressed a clear preference for a flexible, multi-modal approach to the delivery of peer support, with this including both online and offline options, synchronous and asynchronous methods, and individual and group settings. These preferences align with previous typologies of peer support for cancer patients [12, 15, 33] and research showing patient satisfaction with complex, multicomponent psychosocial interventions [37]. Participants cited both practical and personal reasons for desiring a combination of modalities. For example, rural participants emphasised the importance of online support for accessibility, while also appreciating benefits like anonymity. Previous research supports these findings, showing that rural cancer survivors value digital technology approaches to their care, especially telemedicine [38]. Future peer support should therefore aim to offer a flexible, multi-modal program and further research should explore the efficacy of individual components of such a complex intervention.

Participants argued that peer support is useful at all stages of a cancer journey, supporting guidelines that indicate that psychosocial care should be available from initial diagnosis, through treatment, and into survival and palliative care [39–41]. Participants suggested that the time of diagnosis was a particularly difficult time for patients with a rare cancer, who did not have access to good information about their prognosis. Peer support was therefore deemed to be particularly helpful at this phase of illness, and suggests a need for healthcare practitioners to signpost newly diagnosed patients, or patients who are struggling to receive a diagnosis, to peer support services. This is supported by previous research which found that patients chose when and how to engage with peer support, with treatment decision points deemed a particularly salient time for engaging with peer support [34].

Participants also discussed the challenges of peer support. Both healthcare providers and people with rare cancer expressed concerns about exposure to misinformation, the emotional impact of hearing others’ cancer stories (which could lead to both pessimism and unrealistic optimism), interpersonal issues, and questions about the acceptability and feasibility of peer support. These concerns are supported by a review of qualitative research, which identified challenges in cancer support groups for instance confronting the suffering of others, divergent information needs, distressing group dynamics, and difficulties related to leadership and sustainability [24]. Future research should aim to elucidate strategies for overcoming these challenges, including through review of pre-existing peer support services.

### Study strengths and limitations

This study recruited a diverse group of participants, including patients with a rare cancer and healthcare providers, ensuring a comprehensive range of perspectives. By specifically targeting patients with a rare cancer living in rural, regional, and remote areas, it addressed the needs of a population often underrepresented and underserved in research. The use of purposive maximum variation sampling further enhanced the diversity by including participants with varied backgrounds in terms of age and gender. In particular, the study benefitted from excellent representation of Aboriginal and Torres Strait Islander people, a group often under-represented in health research [42]. This is important given that Aboriginal and Torres Strait Islander people have both a higher incidence rate of cancer than non-Aboriginal and Torres Strait Islanders, as well as an increased mortality rate [43]. Moreover, there is evidence emerging that the peer support model is both appropriate and beneficial for Aboriginal and Torres Strait Islander cancer patients [44, 45].

The study’s collaborative recruitment approach, partnering with Rare Cancers Australia, was also a significant strength. Rare Cancer Australia’s extensive network facilitated the recruitment of a sufficient number of participants, increasing the study’s reach and credibility among potential participants. The use of multiple data collection methods, combining focus groups and individual interviews, allowed for flexibility and enriched the data quality. Capturing both collective and individual perspectives provided a more nuanced understanding of participants’ experiences and views.

However, this study is not without limitations. The recruitment process relied on participants volunteering to contact the research team, thereby introducing self-selection bias. Moreover, given that participants were recruited through an existing non-government organisation supporting people with rare cancers (i.e., Rare Cancers Australia), those who chose to participate were already linked into both peer and information access support. The application of the results to the broader population of people diagnosed with rare cancer is unclear and future research should aim to collect data from people not utilising existing rare cancer support services. Furthermore, the study excluded patients who did not speak English comfortably, or who self-identified as having a serious psychiatric illness. This exclusion criterion resulted in the omission of important groups of people with rare cancer, with these people likely to experience more isolation and have even greater support needs. The observations provided by healthcare providers supporting people with a rare cancer were important contributions to the study, notwithstanding the small sample size and limited diversity. With no healthcare providers identifying as Aboriginal or Torres Strait Islander and a small number of different roles represented, the data from this group might not fully capture the range of views necessary for comprehensive service implementation.

## Conclusions

In this study it was confirmed that several aspects of peer support were described as important for patients with a rare cancer living in rural, regional, and remote settings. Peers provided useful access to information, a crucial need of people diagnosed with rare cancer, particularly at the diagnosis stage. Additionally, participants wanted to hear from patients further along in treatment, with this viewed as particularly important for patients with a rare cancer, who often have a limited prognosis. Practical considerations were also noted; the rural location of participants resulted in a preference for online or other remote methods of peer support delivery.

Ultimately, healthcare providers and people with rare cancer emphasised the need for a flexible approach to the development and delivery of peer support. This included being adaptive in considering who a peer is, as well as offering complex, multicomponent and multimodal interventions that meet the varied and fluctuating needs of patients with a rare cancer living in rural, regional and remote communities. Future research should aim to build on the foundations of this study by co-designing a multimodal peer support intervention with patients with a rare cancer living in rural, regional and remote communities.

## Electronic supplementary material

Below is the link to the electronic supplementary material.

Supplementary Material 1

Supplementary Material 2

## Data Availability

The datasets generated and analysed during the current study are not publicly available due to risk of reidentification of participants.

## References

[1] Gatta G, Van Der Zwan JM, Casali PG, Siesling S, Dei Tos AP, Kunkler I, et al. Rare cancers are not so rare: the rare cancer burden in Europe. Eur J Cancer. 2011;47(17):2493–511. 22033323 10.1016/j.ejca.2011.08.008

[2] de Heus E, Duijts SFA, van der Zwan JM, Kapiteijn E, van Dijkum EJMN, van Herpen CML, et al. The gap between rare and common cancers still exists: results from a population-based study in the Netherlands. Eur J Cancer. 2022;167:103–11. 35421702 10.1016/j.ejca.2022.03.001

[3] de Heus E, van der Zwan JM, Husson O, Frissen AR, van Herpen CML, Merkx MAW, et al. Unmet supportive care needs of patients with rare cancer: A systematic review. Eur J Cancer Care. 2021;30(6):e13502. 10.1111/ecc.13502 34409667

[4] Pillai RK, Jayasree K. Rare cancers: challenges & issues. Indian J Med Res. 2017;145(1):17–27. 28574010 10.4103/ijmr.IJMR_915_14 PMC5460568

[5] DeSantis CE, Kramer JL, Jemal A. The burden of rare cancers in the united States. Cancer J Clin. 2017;67(4):261–72. 10.3322/caac.21400 28542893

[6] Low CE, Loke S, Pang GE, Sim B, Yang VS. Psychological outcomes in patients with rare cancers: a systematic review and meta-analysis. Eclinicalmedicine. 2024;72. 10.1016/j.eclinm.2024.102631 PMC11079476 38726223

[7] Van Der Kruk SR, Butow P, Mesters I, Boyle T, Olver I, White K et al. Psychosocial well-being and supportive care needs of cancer patients and survivors living in rural or regional areas: A systematic review from 2010 to 2021. Support Care Cancer. 2022;30:1–44. 10.1007/s00520-021-06440-1 PMC8364415 34392413

[8] Australian Insittute of Health and Welfare. Cancer in Australia 2019. Canberra; 2019.

[9] Charlton M, Schlichting J, Chioreso C, Ward M, Vikas P. Challenges of rural cancer care in the united States. Oncol (Williston Park). 2015;29(9):633–40. 26384798

[10] Sariman JA, Harris NM, Harvey D, Sansom-Daly UM. The experiences of young people living with Cancer in regional and remote Australia: A qualitative study. Australian Social Work. 2022;75(2):205–18.

[11] Goodwin B, Zajdlewicz L, Stiller A, Johnston EA, Myers L, Aitken JF, et al. What are the post-treatment information needs of rural cancer survivors in Australia? A systematic literature review. Psycho-oncology. 2023;32(7):1001–12. 37248643 10.1002/pon.6169

[12] Dunn J, Steginga SK, Rosoman N, Millichap D. A review of peer support in the context of cancer. J Psychosoc Oncol. 2003;21(2):55–67.

[13] Mead S, Hilton D, Curtis L. Peer support: a theoretical perspective. Psychiatr Rehabil J. 2001;25(2):134. 11769979 10.1037/h0095032

[14] Darby Penney MLS. Defining peer support: Implications for policy, practice, and research. Advocates for human potential, inc. 2018.

[15] Hoey LM, Ieropoli SC, White VM, Jefford M. Systematic review of peer-support programs for people with cancer. Patient Educ Couns. 2008;70(3):315–37. 18191527 10.1016/j.pec.2007.11.016

[16] Boyes A, Turon H, Hall A, Sanson-Fisher R. What models of peer support are most appealing to cancer patients? A cross-sectional survey. Asia-Pac J Clin Oncol. 2016;12(Supplement 6):7.26919390

[17] Boyes A, Turon H, Hall A, Watson R, Proietto A, Sanson-Fisher R. Preferences for models of peer support in the digital era: A cross-sectional survey of people with cancer. Psycho-oncology. 2018;27(9):2148–54. 29808504 10.1002/pon.4781

[18] Dennis C-L. Peer support within a health care context: a concept analysis. Int J Nurs Stud. 2003;40(3):321–32. 12605954 10.1016/s0020-7489(02)00092-5

[19] Park HY, Kim MJ, Kim JY, Kim S, Choi JY, Kim JH, et al. Could peer support programs be a good resource for managing the unmet needs of cancer patients? J Cancer Educ. 2019;34:950–7. 30091013 10.1007/s13187-018-1399-4 PMC6785582

[20] Graham-Wisener L, Dempster M. Peer advice giving from posttreatment to newly diagnosed esophageal cancer patients. Dis Esophagus. 2017;30(10). 10.1093/dote/dox089 28859397

[21] Mead S, MacNeil C. Peer support: what makes it unique. Int J Psychosocial Rehabilitation. 2006;10(2):29–37.

[22] Ussher J, Kirsten L, Butow P, Sandoval M. What do cancer support groups provide which other supportive relationships do not? The experience of peer support groups for people with cancer. Soc Sci Med. 2006;62(10):2565–76. 16303220 10.1016/j.socscimed.2005.10.034

[23] Docherty A. Experience, functions and benefits of a cancer support group. Patient Educ Couns. 2004;55(1):87–93. 15476994 10.1016/j.pec.2003.08.002

[24] Jablotschkin M, Binkowski L, Markovits Hoopii R, Weis J. Benefits and challenges of cancer peer support groups: A systematic review of qualitative studies. Eur J Cancer Care. 2022;31(6):Jan–17. 10.1111/ecc.13700 36104303

[25] Allman-Payne P, Rice J. Equitable access to diagnosis and treatment for individuals with rare and less common cancers, including neuroendocrine cancer. Canberra: Senate Printing Unit;: Parliament House; 2024.

[26] Malterud K, Siersma VD, Guassora AD. Sample size in qualitative interview studies: guided by information power. Qual Health Res. 2016;26(13):1753–60. 26613970 10.1177/1049732315617444

[27] Lambert SD, Loiselle CG. Combining individual interviews and focus groups to enhance data richness. J Adv Nurs. 2008;62(2):228–37. 18394035 10.1111/j.1365-2648.2007.04559.x

[28] Katz-Buonincontro J. Combining interviews and focus groups and mixing methods. 2022.

[29] VCCC Alliance. 2024 VCCC Alliance cost model for consumer sitting fees and hourly rate remuneration 2024 [Available from: https://vcccalliance.org.au/assets/about-vccc/Consumer-engagement/Documents/2023/2024-VCCC-Alliance-Cost-Model-v2.pdf

[30] Lumivero. NVivo (Version 14). 2023.

[31] Braun V, Clarke V. Using thematic analysis in psychology. Qualitative Res Psychol. 2006;3(2):77–101.

[32] Braun V, Clarke V. Reflecting on reflexive thematic analysis. Qualitative Res Sport Exerc Health. 2019;11(4):589–97.

[33] Boyes A, Turon H, Hall A, Sanson-Fisher R. Cancer patients’ preferences for models of peer support in the digital age: A crosssectional survey. Psycho-oncology. 2017;26(Supplement 3):68. 10.1002/pon.4781 29808504

[34] Sheridan R, McCaughan D, Hewison A, Roman E, Smith A, Patmore R, et al. Experiences and preferences for psychosocial support: a qualitative study exploring the views of patients with chronic haematological cancers. BMJ Open. 2023;13(8):e070467. 37597866 10.1136/bmjopen-2022-070467 PMC10441118

[35] Crane DA, Lepicki T, Knudsen K. Unique and common elements of the role of peer support in the context of traditional mental health services. Psychiatr Rehabil J. 2016;39(3):282. 27159112 10.1037/prj0000186

[36] Watson E. The mechanisms underpinning peer support: a literature review. J Mental Health. 2017;28(6):677–688.. 10.1080/09638237.2017.1417559 29260930

[37] Taylor E, Ismail S, Hills H, Ainsworth S. Multicomponent psychosocial support for newly diagnosed cancer patients: participants’ views. Int J Palliat Nurs. 2004;10(6):287–95. 15284624 10.12968/ijpn.2004.10.6.13271

[38] Morris BB, Rossi B, Fuemmeler B. The role of digital health technology in rural cancer care delivery: A systematic review. J Rural Health. 2022;38(3):493–511. 34480506 10.1111/jrh.12619 PMC8894502

[39] Cancer Australia. All about multidisciplinary care. 2013.

[40] Cancer Australia. Clinical guidance for responding to suffering in adults with cancer. Cancer Australia: Syd. 2014.

[41] de Heus E, van de Camp K, Driehuis E, van der Zwan JM, van Herpen CML, Merkx MAW, et al. The solitary versus supported experience: care inequality between rare and common cancer patients. Psycho-oncology. 2023;32(11):1667–74. 37698502 10.1002/pon.6216

[42] Jennings W, Spurling G, Shannon B, Hayman N, Askew D. Rapid review of five years of aboriginal and Torres Strait Islander health research in Australia–persisting under-representation of urban populations. Aust N Z J Public Health. 2021;45(1):53–8. 33522668 10.1111/1753-6405.13072

[43] Australian Institute of Health and Welfare. Cancer in Aboriginal & Torres Strait Islander people of Australia. Australian Government. 2018.

[44] Cuesta-Briand B, Bessarab D, Shahid S, Thompson SC. Connecting tracks’: exploring the roles of an aboriginal women’s cancer support network. Health Soc Care Commun. 2016;24(6):779–88. 10.1111/hsc.12261 26099647

[45] Cuesta-Briand B, Bessarab D, Shahid S, Thompson SC. Addressing unresolved tensions to build effective partnerships: lessons from an aboriginal cancer support network. Int J Equity Health. 2015;14:1–0. 26537924 10.1186/s12939-015-0259-7 PMC4634592

