# Supplementary material for: What do patients with a rare cancer living in rural, regional or remote areas and stakeholders want from a peer support program? A qualitative study
Source: BMC Cancer. 2025 Feb 25;25:352. doi: 10.1186/s12885-025-13782-0 (PMC11863523; doi:10.1186/s12885-025-13782-0)
Supplement: Supplementary file 1 — Supplementary Material 1 [file 12885_2025_13782_MOESM1_ESM.docx]

# Appendix A - Patient topic guide

1. What is your experience with peer-led supportive care?
   1. What should / could peer support do differently than it does currently?
   2. What needs does it not meet?
2. Do you think peer support would / is / could be helpful?
   1. What can peer support offer you?
   2. Is it helpful for people living in rural / remote areas?
   3. When would it be helpful? (e.g. phases of illness)
   4. What would be unhelpful in peer support?
   5. Reciprocity
3. Who would you consider a peer? *(prompts: same diagnosis, same demographic factors, same phase of illness)?*
   1. Would this be the same person throughout your illness trajectory?
   2. Someone with the same side effects / symptoms?
   3. What are some of the traits that are important to you in a peer
4. What would you like to see included in a peer support intervention for rural people with a rare cancer?
   1. How long do you want to take part in a peer support group?
   2. Are there any specific issues you would like peer support to help with? *Prompt: sexuality, fertility?*
   3. Do you want to meet with the same group each time or a different group?
   4. Do you want groups to morph over time?
   5. Format (group vs 121) – how many in a group?
   6. Structured vs unstructured
   7. Content – what do you want to achieve from it
   8. Anything specific to rare cancers?
   9. Anything specific to rural?
5. Anything else you would like to mention
