# Supplementary material for: What do patients with a rare cancer living in rural, regional or remote areas and stakeholders want from a peer support program? A qualitative study
Source: BMC Cancer. 2025 Feb 25;25:352. doi: 10.1186/s12885-025-13782-0 (PMC11863523; doi:10.1186/s12885-025-13782-0)
Supplement: Supplementary file 2 — Supplementary Material 2 [file 12885_2025_13782_MOESM2_ESM.docx]

# Appendix B - Healthcare provider topic guide

1. Tell me about your experience working with people with a rare cancer
   1. Tell me about your experiences in rural areas
2. What sort of psychosocial support does your service currently offer for rare cancer / rural patients?
   1. What works well?
   2. What doesn’t work well?
3. Tell me about your experience / knowledge / opinion of peer support for cancer
   1. Would peer support work for this group?
4. What would you like to see included in a peer support intervention for rural people with a rare cancer?
5. Anything else you would like to mention
